# Augmented cardiac growth hormone signaling in circadian-disrupted cardiomyopathy

**DOI:** 10.3389/fphar.2026.1982446

**Published:** 2026-10-20

**Authors:** Dina Ibrahim, Ravi Sonkar

**Affiliations:** 1 Department of Surgery, Division of Vascular Surgery, Washington University in St. Louis, St. Louis, MO, United States; 2 Department of Medicine, Division of Cardiology, Washington University School of Medicine, St. Louis, MO, United States

**Keywords:** cardiac hypertrophy, cardiomyopathy, chronopharmacology, circadian clock, growth hormone, pegvisomant, REV-ERBα, STAT5

## Abstract

Circadian disruption, including rotating shift work, social jet lag, and chronic sleep disturbance, is associated with increased cardiovascular risk and may promote cardiac remodeling. The molecular mechanisms connecting clock disruption to structural heart disease remain incompletely defined. This minireview advances the hypothesis that the growth hormone, GH, axis is an underrecognized endocrine mediator. GH secretion is time dependent, with most daily secretion occurring during early nocturnal sleep-in humans. Cardiomyocytes express GH receptors, and GH can activate Janus kinase 2, signal transducer and activator of transcription 5, phosphoinositide 3 kinase and Akt, and mitogen activated protein kinase and extracellular signal regulated kinase pathways that influence cardiac growth and metabolism. We propose that the cardiomyocyte molecular clock restricts GH responsiveness to circadian phases. REV ERBα is a plausible component of this restraint, but direct cardiomyocyte evidence is lacking. Circadian disruption may increase or mistime myocardial GH signaling, promoting hypertrophy, fibrosis, and metabolic inflexibility. The strongest support comes from cardiomyocyte specific *Bmal1* deficient mice, which show increased GH induced cardiac STAT5 phosphorylation, increased cardiac *Igf1* expression, and remodeling. Reduction of GH receptor gene dosage partially attenuates this phenotype, supporting a causal contribution of cardiac GH signaling. Nevertheless, genetic clock ablation is not equivalent to environmental circadian misalignment in shift work or chronic sleep disruption. We critically evaluate the components of this proposed clock-GH-heart axis, identify unresolved questions, and outline experiments and chronopharmacological strategies to test whether restoring temporal control of GH signaling can prevent pathological remodeling while preserving physiological GH action.

## 1 Introduction

Approximately 15%–20% of the workforce in industrialized countries performs shift work. Meta-analyses indicate that long-term rotating-shift work is associated with a 20%–40% excess risk of coronary heart disease and incident heart failure, even after adjustment for smoking, obesity, and hypertension (Torquati et al., 2018; Vetter et al., 2016). These observations suggest that circadian disruption injures the heart through mechanisms that extend beyond conventional lifestyle-related risk factors. However, the molecular pathways that translate disrupted biological timing into structural heart disease remain incompletely defined.

Cardiomyocytes contain a cell-autonomous molecular clock that coordinates cardiac physiology across the 24-h day. BMAL1:CLOCK heterodimers drive *E*-*box*-dependent transcription of *Per* and *Cry* genes, whose protein products subsequently feedback to repress BMAL1: CLOCK activity. *REV-ERBα/β* and *RORα* form a stabilizing accessory loop by acting through *ROREs* (ROR response elements) to regulate *Bmal1* transcription (Young et al., 2014; Takahashi, 2017). Approximately 8%–13% of the cardiac transcriptome oscillates over 24 h, governing metabolic substrate selection, contractile protein turnover, and stress responses (Young et al., 2014). Consistent with the importance of this clock, cardiomyocyte-specific deletion of *Bmal1* causes age-dependent dilated cardiomyopathy in mice, while environmental circadian-disruption protocols, including chronic phase shifts cause cardiac hypertrophy and fibrosis in wild-type animals (Young et al., 2014; Martino et al., 2008).

Most discussions of circadian cardiomyopathy focus on metabolic dysregulation, NF-κB-driven inflammation, or hemodynamic abnormalities such as loss of nocturnal blood pressure dipping. GH signaling in the heart is discussed almost exclusively in the acromegaly literature. Three facts, however, converge and have not been assembled into a cardiac-specific framework. First, the heart expresses functional GH receptors and responds directly to GH through signaling programs that regulate hypertrophy and fibrosis. Second, GH secretion is strongly circadian-gated, with approximately 70% of daily GH release during early nocturnal sleep (Leproult and Van Cauter, 2010; Van Cauter et al., 1998). Third, circadian disruption alters GH pulse amplitude, timing, and trough concentrations in humans and rodents (Leproult and Van Cauter, 2010; Van Cauter et al., 1998). We therefore propose that the cardiac clock gates GH signaling by restricting GH receptor availability, downstream signal-transduction efficiency, or both, to define circadian windows. Circadian disruption may remove this protective temporal restraint, allowing sustained or mistimed GH pathway activation that drives pathological remodeling.

## 2 The temporal gating hypothesis

The biological outcome of growth factor signaling are determined not only on ligand concentration, but also on timing, duration, and cellular context of exposure. A cardiomyocyte receiving a GH pulse during a permissive anabolic phase may engage adaptive growth programs, whereas exposure to an identical GH pulse during a catabolic, stress-responsive, or contractile phase may elicit a different transcriptional response (Bray et al., 2008). The molecular clock provides this temporal context by rhythmically controlling receptor abundance, intracellular signal-transduction components, chromatin accessibility, and negative feedback regulators. Temporal gating of this kind has been demonstrated for glucocorticoid receptor signaling in liver and for Toll-like receptor 4 responses in macrophages (Caratti et al., 2018). We extend the principle here to cardiac GH signaling.

The hypothesis comprises five testable nodes (Figure 1). (i) We therefor propose that clock-controlled REV-ERBα periodically represses the transcription of GH signaling components in cardiomyocytes, including the GH receptor (*Ghr*) and one or both STAT5 isoforms (*Stat5a* and *Stat5b*). This would create a daily phase of relative GH sensitivity when REV-ERBα activity is low, and a phase of reduced responsiveness when REV-ERBα activity is high. No study has yet demonstrated direct REV-ERBα binding at the *Ghr*, *Stat5a*, or *Stat5b* promoters in cardiomyocytes; this node rests entirely on analogy to hepatic REV-ERBα function and remains the highest-priority target for cardiac chromatin immunoprecipitation experiments. (ii) Clock-controlled *Socs2* expression may establish a parallel negative-feedback rhythm that limits the duration of each GH signal. (iii) Circadian disruption flattens REV-ERBα oscillation and *Socs2* rhythmicity, de-repressing GH pathway genes across the entire cycle. (iv) The net effect would be heightened GH pathway responsiveness that, if combined with redistributed or elevated GH pulses, could produce a functional analog of chronic GH excess at the receptor and transduction level. Two points of precision are essential here. Increased responsiveness to a GH stimulus (higher peak pSTAT5 per unit GH) is not the same as constitutive, tonic pathway activation; the distinction matters because their downstream consequences for gene expression, hypertrophy, and fibrosis may differ in magnitude and pattern. The available data (Section 3.2) demonstrate the former; the latter is inferred but unproven. (v) This heightened and temporally disorganized activation is predicted to drive pathological hypertrophy, fibrosis, and metabolic remodeling rather than physiological cardiac growth.

**FIGURE 1:**
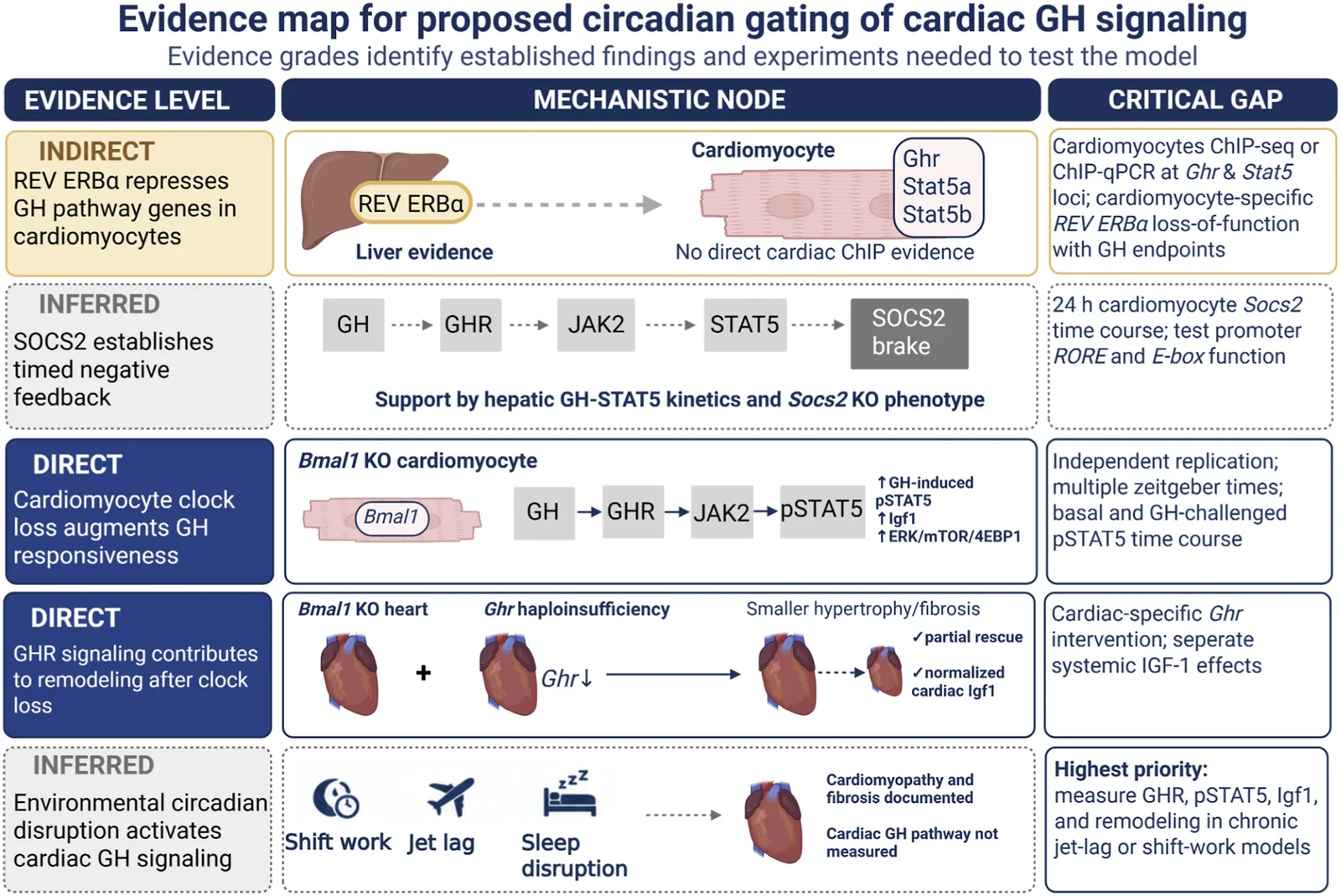
Evidence map for proposed circadian gating of cardiac growth hormone signaling. This figure summarizes the evidence supporting five mechanistic nodes linking cardiomyocyte circadian clock disruption to augmented GH signaling and pathological cardiac remodeling. Evidence is classified as direct, indirect, or inferred. Direct evidence indicates findings demonstrated in cardiomyocytes or hearts using the stated genetic manipulation. Indirect evidence indicates relevant observations from another tissue or related experimental model. Inferred evidence identifies biologically plausible mechanisms that have not been tested in the cardiac context. The first node is indirect: hepatic studies support REV ERBα dependent transcriptional regulation, but direct repression of *Ghr*, *Stat5a*, or *Stat5b* by REV ERBα has not been demonstrated in cardiomyocytes. The second node is inferred: SOCS2 is a GH STAT5 responsive negative feedback regulator, but its circadian expression and direct clock control in cardiomyocytes remain unknown. The third and fourth nodes are supported by direct evidence from cardiomyocyte specific *Bmal1* knockout mice, in which GH stimulation increases cardiac STAT5 phosphorylation, *Igf1* expression, and downstream ERK, mTOR, and 4EBP1 signaling. Global *Ghr* haploinsufficiency partially attenuates cardiomyopathy and normalizes cardiac *Igf1* expression, supporting a contributory role for GH receptor signaling in remodeling after cardiomyocyte clock loss. The fifth node remains inferred: environmental circadian disruption, including shift work, chronic jet lag, and sleep disruption, is associated with cardiac fibrosis and cardiomyopathy, but cardiac GH pathway activation has not been measured in these models. Critical experiments required to test each node are shown in the right column. Solid arrows denote experimentally established relationships. Gray dashed arrows indicate proposed, indirect, or untested mechanisms. GH, growth hormone; GHR, growth hormone receptor; JAK2, Janus kinase 2; STAT5, signal transducer and activator of transcription 5; SOCS2, suppressor of cytokine signaling 2; REV ERBα, nuclear receptor subfamily 1 group D member 1; *Bmal1*, brain and muscle ARNT like 1; *Igf1*, insulin like growth factor 1; ChIP, chromatin immunoprecipitation; KO, knockout; ZT, zeitgeber time; ECM, extracellular matrix. Created in BioRender. Arif, B. (2026) https://BioRender.com/2m28ssd.

A further distinction must be kept in view throughout. Cardiomyocyte-specific *Bmal1* deletion abolishes the molecular clock entirely, eliminating oscillation of all clock-controlled outputs. Shift work, social jet lag, and chronic sleep disruption, by contrast, misalign the clock, altering the phase, amplitude, and inter-organ synchrony of circadian oscillations without necessarily abolishing them (Martino et al., 2008; Durgan et al., 2011). Genetic ablation therefore represents a ceiling estimate of what clock perturbation can do to GH signaling, not a direct model of what environmental disruption does do. Findings from the CBK model establish biological plausibility and identify mechanism; translation to the clinical setting requires environmental disruption models that misalign without abolishing the clock, as discussed in Section 3.3.

We classify the evidence supporting each node as direct, indirect, or inferred. Direct evidence denotes a finding demonstrated in cardiomyocytes using the precise genetic, pharmacological, or circadian manipulation under consideration. Indirect evidence denotes a finding from another tissue, cell type, or related perturbation that supports but does not establish the proposed cardiac mechanism. Inferred evidence is biologically plausible and logically derived from established principles but has not yet been experimentally tested in the cardiac context. Table 1 summarizes each node with its evidence grade, experimental model and tissue, type of circadian disruption, and the experiment needed to resolve it.

**TABLE 1:** Evidence map for the proposed temporal gating of cardiac growth hormone signaling by the circadian clock. Each mechanistic node of the hypothesis (Figure 1) is graded as *Direct* (demonstrated in cardiomyocytes using the specific manipulation under consideration), *Indirect* (demonstrated in other tissues or with related manipulations that support but do not establish the proposed cardiac mechanism), or *Inferred* (biologically plausible and logically derived from established principles but not yet experimentally tested in the cardiac context).

Hypothesis node	Grade	Model/Strain	Tissue/Cell type	Type of circadian disruption	Key evidence	Critical gap
REV-ERBα represses GH pathway genes in cardiomyocytes	Indirect	*C57BL/6J* (*Rev-erbα* studies) CBK mice (*αMHC-Cre × Bmal1^fl/fl^*)	Liver (Rev-erbα ChIP) Whole heart (CBK GH sensitivity)	Genetic *(Rev-erbα/β* DKO in liver *Bmal1* KO in cardiomyocytes)	REV-ERBα represses metabolic and growth genes genome-wide in liver (Cho et al., 2012); CBK hearts show augmented GH sensitivity (Sonkar et al., 2022)	No cardiac ChIP-seq at *Ghr* or Stat5 loci; no cardiomyocyte-specific Rev-erbα KO with GH endpoints
*Socs2* oscillates in cardiomyocytes under clock control	Inferred	*Socs2* KO mice (*C57BL/6J*) Hepatic GH pulse studies	Liver No cardiac data	N/A (inferred from hepatic GH-STAT5 kinetics)	SOCS2 is a GH-STAT5 target; *Socs2* KO phenocopies GH excess (Greenhalgh et al., 2002); hepatic SOCS2 follows GH pulse timing	No time-course *Socs2* data in cardiomyocytes; promoter RORE/E-box analysis absent
Clock disruption augments cardiac GH responsiveness and STAT5 activation	Direct	CBK mice (*αMHC-Cre × Bmal1^fl/fl^* *C57BL/6J* background)	Whole heart lysates; cardiac-specific effect confirmed vs. liver	Genetic (cardiomyocyte-specific *Bmal1* deletion)	CBK hearts: ↑ pSTAT5 after GH injection, ↑ *Igf1* mRNA, activated pERK/pmTOR/p4EBP1; cardiac-specific (Sonkar et al., 2022)	Single laboratory; single genetic model; single time-point GH challenge; no ZT time-course for pSTAT5
Augmented GH signaling contributes causally to circadian cardiomyopathy	Direct	CBK mice × global *Ghr* heterozygous deletion	Whole heart (echo, histology, mRNA)	Genetic (*Bmal1* KO + *Ghr* haploinsufficiency)	*Ghr* haploinsufficiency normalizes cardiac *Igf1* and partially attenuates CBK cardiomyopathy (Sonkar et al., 2022)	Global (not cardiac-specific) *Ghr* deletion; incomplete rescue; cannot exclude systemic IGF-1 reduction; not independently replicated
Environmental circadian disruption activates cardiac GH signaling	Inferred	Golden hamsters (chronic phase shifts) Wistar rats (forced activity) Humans (forced desynchrony)	Heart (histology, transcriptome in rats (Trott et al., 2022)) Serum (GH profiles in humans)	Environmental (12-h phase reversals; forced rest-phase activity; forced desynchrony)	Chronic jet lag causes cardiomyopathy (Martino et al., 2008); shift work reprograms cardiac transcriptome and causes fibrosis (Trott et al., 2022); human disruption redistributes GH pulses (Leproult and Van Cauter, 2010; Van Cauter et al., 1998)	No environmental model has measured cardiac GH/IGF-1 parameters; highest-priority experiment

For each node, the experimental model, tissue or cell type, and type of circadian disruption are specified. The critical gap column identifies the experiment most needed to resolve or refute the proposed mechanism. CBK, cardiomyocyte-specific *Bmal1* knockout; DKO, double knockout; ChIP, chromatin immunoprecipitation; ZT, zeitgeber time; echo, echocardiography; LV, left ventricular.

## 3 Evidence for clock-gated cardiac GH signaling

### 3.1 The hepatic precedent

The liver provides an important precedent for circadian regulation of GH signaling. Hepatic *Ghr* expression, JAK2 abundance, and GH-induced STAT5 activity exhibit time-of-day variation. In rodents, pulsatile GH-STAT5 signaling drives time-dependent transcriptional programs, and REV-ERBα can modulate STAT5-dependent transcriptional output at hepatic gene promoters (Waxman and O’Connor, 2006; Cho et al., 2012). SOCS2, the principal GH-specific negative regulator (whose genetic deletion produces gigantism phenocopying GH excess (Greenhalgh et al., 2002)), is itself a STAT5 transcriptional target and thus follows GH pulse timing. Whether the *Socs2* promoter also contains functional *RORE* elements, which would make it a direct clock target independent of GH input, has not been tested in any tissue. Thus, the hepatic literature demonstrates that GH responsiveness can be regulated at multiple levels; however, the heart is not the liver, and direct cardiac validation is essential.

### 3.2 Direct cardiac evidence from cardiomyocyte-specific *Bmal1* knockout mice

The strongest support for the hypothesis comes from the cardiomyocyte-specific *Bmal1* knockout model (CBK; αMHC-Cre × *Bmal1* ^*fl/fl*^). Sonkar et al. showed that CBK hearts exhibit augmented GH-induced STAT5 phosphorylation relative to floxed controls, with approximately two-fold higher *Igf1* mRNA levels and activation of downstream effectors including phosphorylated ERK, mTOR, and 4EBP1 (Sonkar et al., 2022). This increase in GH responsiveness was cardiac-specific; livers from the same animals showed no corresponding change, indicating that the cardiomyocyte clock selectively restricts local GH responsiveness rather than reflecting a systemic alteration.

A critical nuance: these data demonstrate that clock-disrupted cardiomyocytes mount a larger acute response to GH, not that JAK2-STAT5 signaling is constitutively active in CBK hearts in the absence of GH stimulation. The study measured pSTAT5 after exogenous GH injection at a single time point; whether CBK hearts exhibit elevated basal pSTAT5 throughout the circadian cycle, which would constitute true sustained activation, was not assessed. Future studies sampling pSTAT5 across multiple zeitgeber times, with and without GH challenge, are needed to distinguish increased sensitivity from tonic activation.

The causal test is equally informative. Genetic deletion of one *Ghr* allele normalized cardiac *Igf1* expression in CBK mice and was associated with partial attenuation of the cardiomyopathic phenotype, including reduced hypertrophy and slowed fibrosis progression (Sonkar et al., 2022). This result moves the GH pathway from correlation to contribution: augmented cardiac GH responsiveness is not merely a bystander but actively promotes remodeling in the setting of clock disruption. Two caveats deserve mention. The *Ghr* haploinsufficiency was global, not cardiac-specific, so a contribution from reduced systemic IGF-1 cannot be excluded. In addition, the rescue was partial, indicating that GH signaling is one of several pathways through which clock loss damages the heart.

A further interpretive layer concerns the downstream readout. The elevated *Igf1* mRNA in CBK hearts indicates that augmented GHR-JAK2-STAT5 signaling drives local IGF-1 production, but some of the observed downstream activation, particularly of PI3K-Akt and mTOR, could reflect autocrine/paracrine IGF-1 acting through IGF-1 receptors rather than direct GHR-mediated signaling. IGF-1R shares substantial downstream overlap with both the insulin receptor and GHR itself; moreover, cardiomyocytes express IGF-1R/IR hybrid receptors whose signaling properties differ from either homodimer (Sonkar et al., 2022; Belfiore et al., 2009). The *Ghr* haploinsufficiency experiment suggests that GHR is upstream because reducing GHR gene dosage normalized *Igf1* expression and attenuated the phenotype, but it does not exclude an independent contribution of IGF-1R-mediated signaling to the remodeling process. Dissecting these contributions would require cardiac-specific *Igf1r* deletion or pharmacological IGF-1R blockade (e.g., with linsitinib or NVP-AEW541) in the CBK model, experiments that have not been performed.

### 3.3 Environmental disruption models: the missing measurement

Chronic jet-lag protocols produce cardiomyopathy in wild-type hamsters and mice after 48–60 weeks of weekly 12-h phase reversals (Martino et al., 2008). In a rat forced-activity shift-work model (8 h of enforced wakefulness during the rest phase, 5 days per week for 5 weeks), Trott et al. demonstrated profound reprogramming of the cardiac cycling transcriptome, with altered rhythmic gene expression, upregulation of extracellular matrix and inflammatory genes, and histologically confirmed cardiac fibrosis (Belfiore et al., 2009; Che et al., 2024; Trott et al., 2022). Neither the chronic jet-lag paradigm nor the forced-activity model measured cardiac GH pathway parameters. This omission constitutes the single largest gap in the evidence base.

The gap matters for a specific reason beyond simple incompleteness. Environmental models misalign the clock rather than abolishing it. They may therefore produce mistimed rather than constitutive GH pathway activation, a biologically distinct and clinically more relevant scenario than genetic ablation. The CBK model eliminates all clock-controlled transcriptional oscillation, which should maximally de-repress GH pathway genes at all time points. Environmental disruption, by contrast, may phase-shift or dampen REV-ERBα oscillations without eliminating them, producing periodic rather than continuous windows of excess GH responsiveness. The downstream consequences, whether partial versus complete loss of temporal gating leads to the same degree of hypertrophy and fibrosis, or to qualitatively different remodeling patterns, are unknown. Human forced-desynchrony studies show blunted nocturnal GH surges, redistribution of GH pulses across the 24-h cycle, and elevated trough levels under circadian misalignment (Leproult and Van Cauter, 2010; Van Cauter et al., 1998). Clinical implications should therefore be considered provisional: shift work and sleep disruption are best modeled as circadian misalignment, not as cardiomyocyte clock ablation, and cardiac GH-pathway measurements in environmentally disrupted humans are currently absent.

## 4 Pharmacological strategies

The pharmacological implications of the hypothesis fall into two categories: agents that suppress GH signaling directly and agents that restore circadian gating. For a cardiovascular pharmacology audience, the translational question is whether either approach, or a timed combination, can prevent or reverse the cardiac remodeling associated with circadian disruption.

### 4.1 GH axis suppression: somatostatin analogues and pegvisomant

Octreotide and lanreotide are somatostatin receptor subtype 2 (SST2) agonists that suppress pituitary GH secretion. In acromegaly, six to 12 months of biochemical control reduces left ventricular mass index by 5–15% and improves diastolic function, with modest but consistent effect sizes across trials (Colao et al., 2011; Pivonello et al., 2017). Pasireotide, a multi-receptor agonist with higher affinity for SST5 than SST2, achieves biochemical control in octreotide-resistant patients but carries substantial hyperglycemia risk due to SST5-mediated suppression of insulin secretion (Colao et al., 2014). That side effect is particularly concerning a shift-work population already predisposed to insulin resistance from circadian disruption.

The timing problem in circadian cardiomyopathy differs fundamentally from acromegaly. In acromegaly, GH is tonically elevated, so suppression at any time of day is beneficial. Under circadian disruption, GH remains pulsatile but mis-timed; the ideal intervention would eliminate ectopic or excess pulses while preserving physiological nocturnal release, which supports cardiac repair and regeneration. Somatostatin analogues suppress all GH secretion without temporal selectivity. Because GH deficiency is itself cardiotoxic (producing wall thinning, impaired contractility, and increased cardiovascular mortality in hypopituitary patients (Maison and Chanson, 2003)), the therapeutic window may be narrow. Short-acting octreotide (plasma half-life approximately 90 min subcutaneously) administered at the time of peak ectopic GH release is one chronopharmacological strategy, but it requires knowledge of each patient’s disrupted GH secretion profile.

Pegvisomant offers a mechanistically cleaner approach. This PEGylated GH analogue competitively antagonizes the GH receptor without activating it and normalizes serum IGF-1 in approximately 90% of acromegaly patients at doses of 10–30 mg per day given subcutaneously (Trainer et al., 2000). Unlike somatostatin analogues, pegvisomant blocks the receptor regardless of when GH pulses arrive, eliminating the chronopharmacological uncertainty. Pituitary GH output rises because IGF-1-mediated negative feedback is interrupted, but this increase is pharmacologically irrelevant when the receptor is blocked. Hepatic transaminase elevations occur in 5%–10% of treated patients, are generally mild and reversible, and cardiac safety data from acromegaly registries have been reassuring (Pivonello et al., 2017). For hypothesis testing, the key preclinical experiment is straightforward: pegvisomant versus vehicle in mice undergoing a chronic jet-lag protocol, with echocardiographic, histological, and molecular endpoints at 24 and 48 weeks.

### 4.2 Clock-targeting small molecules

SR9009 and SR9011 bind the ligand-binding domain of REV-ERBα/β, enhancing NCoR/HDAC3 corepressor recruitment and amplifying transcriptional repression (Solt et al., 2012). Within the framework of this review, REV-ERB agonists should restore repression of GH pathway genes even when endogenous REV-ERBα rhythmicity is compromised. Published cardiac data are encouraging: SR9009 reduced fibrosis and improved function in a murine pressure-overload model (Stujanna et al., 2017). Neither that study nor any other measured GH signaling endpoints after REV-ERB agonist treatment. Several pharmacological limitations must be acknowledged. SR9009 has a plasma half-life under 1 hour in mice and poor oral bioavailability. Dierickx et al. demonstrated that SR9009 retains metabolic effects in *Rev-erbα/β* double-knockout cells, indicating significant off-target activity (Dierickx et al., 2019). More selective REV-ERB agonists are under development but have not reached cardiac testing.

Nobiletin, a polymethoxylated flavone, enhances circadian oscillation amplitude and protects against metabolic syndrome and ischemia-reperfusion injury in rodent models (He et al., 2016; Nohara et al., 2019). Its molecular target is debated (RORα/γ modulation, direct kinase effects, or both), and “enhancing clock amplitude” is pharmacologically imprecise, lacking a clear dose-response relationship for specific molecular targets. Translational advantages include oral bioavailability and a favorable safety profile. CRY stabilizers such as KL001 and its derivatives prolong the negative arm of the clock feedback loop (Hirota et al., 2012), but cardiac data are almost nonexistent. CRY stabilization also lengthens the circadian period, which could worsen internal desynchrony between the cardiac and central clocks.

### 4.3 Toward combination strategies

A rational therapeutic approach would combine a clock stabilizer to restore temporal gating with a timed GH axis modulator to suppress excess signaling during the transition period before clock function recovers. The clock agent addresses the cause of augmented signaling; the GH modulator addresses its consequence. No preclinical data exist for any such combination. Predicted pharmacological interactions deserve consideration: REV-ERB agonists alter hepatic lipid metabolism pathways shared with somatostatin analogue clearance, and the compensatory rise in pituitary GH secretion caused by pegvisomant could itself affect peripheral clock gene expression through STAT5-driven *Per1* induction (Cho et al., 2012; Woelfle et al., 2003; Mad et al., 2014). A further barrier is the absence of validated biomarkers for cardiac-specific circadian disruption in humans; melatonin and cortisol profiles reflect suprachiasmatic nucleus function, not peripheral cardiac clock status.

## 5 Discussion

We propose a clock-GH-heart axis in which the cardiomyocyte molecular clock temporally gates GH signaling. Disruption of this gate may produce heightened GH responsiveness and, when combined with redistributed or elevated GH pulses, functionally excessive GHR-JAK2-STAT5 activation, contributing to cardiac hypertrophy, fibrosis, and metabolic remodeling. The CBK mouse data provide direct evidence for increased cardiac GH sensitivity after genetic clock disruption, and *Ghr* haploinsufficiency partially rescues the phenotype (Sonkar et al., 2022). What this framework explains that purely metabolic or hemodynamic models do not is the coexistence of hypertrophic drive, pro-fibrotic signaling, insulin resistance, and oxidative stress in circadian cardiomyopathy (Lecacheur et al., 2024; Khan et al., 2018). These features parallel acromegalic cardiomyopathy and are simultaneously engaged by sustained GH pathway activation. The current evidence supports enhanced GH responsiveness after cardiomyocyte circadian clock loss, but it does not yet demonstrate direct REV-ERBα regulation of *Ghr*, *Stat5a/b*, or *Socs2* in cardiomyocytes, nor does it distinguish increased acute sensitivity from constitutive tonic activation.

### 5.1 Alternative pathways in circadian cardiomyopathy

The GH axis is unlikely to act in isolation. Circadian disruption activates multiple signaling cascades that converge on pathological remodeling, and the relative contribution of each pathway is unresolved. Che et al. (2024) recently demonstrated that a chronic jet-lag protocol (serial 8-h phase advances every 4 days for 3 months) produced an HFpEF-like phenotype in mice, characterized by cardiac hypertrophy, fibrosis, and diastolic dysfunction, accompanied by attenuation of Clock-dependent soluble guanylate cyclase (sGC) expression and reduced cGMP-PKG1 signaling (Che et al., 2024). Treatment with the sGC stimulator riociguat attenuated these structural changes, providing pharmacological evidence that the sGC-cGMP-PKG1 axis is a functionally relevant mediator of circadian cardiomyopathy independent of GH. Other documented contributors include NF-κB-driven inflammatory signaling, metabolic substrate shifts (reduced fatty acid oxidation, increased glycolysis), and loss of nocturnal blood pressure dipping with consequent hemodynamic overload (Lecacheur et al., 2024; Khan et al., 2018).

Whether these pathways operate independently or converge on shared downstream effectors is an open question. GH-STAT5 signaling, sGC-cGMP-PKG1 signaling, and NF-κB activation target overlapping gene programs governing hypertrophy and fibrosis; for example, PKG1 phosphorylates and inhibits RhoA-dependent fibrotic signaling, while GH-driven STAT5 and mTOR activation promote it. Circadian disruption may thus create a simultaneous enhancement of pro-fibrotic GH signaling and withdrawal of anti-fibrotic cGMP-PKG1 tone. Testing this convergence will require studies measuring both pathways in the same model, ideally comparing GH pathway blockade (pegvisomant), sGC stimulation (riociguat), and their combination in circadian-disrupted animals.

### 5.2 Unresolved questions and priority experiments

Three experiments deserve priority. First, does chronic jet lag increase cardiac GH receptor density or STAT5 phosphorylation in wild-type mice? This would extend the genetic finding to the clinically relevant environmental scenario and determine whether clock misalignment, as opposed to clock abolition, is sufficient to augment cardiac GH responsiveness. Second, does pegvisomant prevent cardiomyopathy in circadian-disrupted animals? A positive result would establish GH receptor activation as a necessary mediator, not merely a correlate. Third, does SR9009 reduce cardiac STAT5 phosphorylation, and is this effect abolished in *Rev-erbα*-deficient mice? This would separate on-target, REV-ERB-dependent GH pathway suppression from the compound’s known off-target effects. A fourth experiment would directly address the sensitivity-versus-sustained-activation distinction: time-series measurement of cardiac pSTAT5 across at least six zeitgeber time points in CBK versus control mice, under both basal conditions and after timed GH injection. If pSTAT5 is elevated only after GH challenge (increased sensitivity), the therapeutic implication is to reduce GH exposure (timing or blocking). If pSTAT5 is elevated basally (tonic activation), the implication is that an intracellular mechanism, independent of ligand, maintains pathway activity, which would require different pharmacological targeting. A fifth experiment would separate GHR-mediated from IGF-1R-mediated contributions: cardiac-specific *Igf1r* deletion or IGF-1R antagonist treatment in the CBK model, with hypertrophy, fibrosis, and pSTAT5/pAkt as endpoints. Additionally, both GH secretion and cardiac GH responsiveness may vary over 24 h; future studies must specify sampling time, use serial time-series designs, and avoid interpreting a single terminal tissue collection as evidence of sustained pathway activation.

### 5.3 Limitations

Several limitations warrant emphasis. Nearly all direct evidence derives from a single laboratory’s work with the CBK model (Sonkar et al., 2022); independent replication is essential. GH physiology differs between rodents and humans in pulsatility patterns and sexual dimorphism. Environmental disruption models, which misalign rather than ablate the clock, have never measured cardiac GH parameters, and this remains the most important data gap. The proposed REV-ERBα-mediated mechanism, while consistent with hepatic precedent, is entirely untested in cardiomyocytes at the chromatin level. A feasible initial clinical study would compare day workers and long-term rotating-shift workers using actigraphy, serial overnight GH sampling or GH-axis surrogates, echocardiographic assessment of left-ventricular mass and diastolic function, and circulating fibrosis markers such as galectin-3, soluble ST2, and procollagen peptides. Clinical translation faces the further obstacle that no validated biomarker exists for cardiac clock disruption severity, and cardiac-specific drug effects are difficult to separate from systemic actions *in vivo*.

## 6 Conclusion

The cardiomyocyte clock functions as a temporal filter that shapes the response to growth and stress signals. Genetic disruption of this clock increases cardiac responsiveness to GH and partially implicates GHR signaling in adverse remodeling. Whether environmental circadian misalignment produces a comparable defect in cardiac GH signaling remains unknown. Defining the circadian GH response curve in normal and disrupted hearts, and testing whether targeted restoration of clock function or GH pathway inhibition prevents remodeling, will determine whether this axis is a therapeutically actionable component of circadian cardiomyopathy.
